# Dislocation substructures in pure aluminium after creep deformation as studied by electron backscatter diffraction

**DOI:** 10.1107/S1600576722005209

**Published:** 2022-07-05

**Authors:** Itziar Serrano-Munoz, Ricardo Fernández, Romeo Saliwan-Neumann, Gaspar González-Doncel, Giovanni Bruno

**Affiliations:** a Bundesanstalt für Materialforschung und -prüfung (BAM), Unter Den Eichen 87, Berlin, 12205, Germany; b Department of Physical Metallurgy, Centro Nacional de Investigaciones Metalúrgicas (CENIM), Avenida de Gregorio del Amo No. 8, Madrid, E-28040, Spain; c Institute of Physics and Astronomy, University of Potsdam, Karl-Liebknecht-Strasse 24–25, Potsdam, 14476, Germany; SLAC National Accelerator Laboratory, Menlo Park, USA

**Keywords:** creep, pure aluminium, electron backscatter diffraction (EBSD), cellular structures, power law and power-law breakdown

## Abstract

In the present work, electron backscatter diffraction was used to determine the microscopic dislocation structures generated during creep (with tests interrupted at the steady state) in pure 99.8% aluminium. This material was investigated at two different stress levels, corresponding to the power-law and power-law breakdown regimes. The results show that the formation of subgrain cellular structures occurs independently of the crystallographic orientation. However, the density of these cellular structures strongly depends on the grain crystallographic orientation with respect to the tensile axis direction, with 〈111〉 grains exhibiting the highest densities at both stress levels. It is proposed that this behaviour is due to the influence of intergranular stresses, which is different in 〈111〉 and 〈001〉 grains.

## Abstract 2

In the present work, electron backscatter diffraction is used to investigate the influence of crystal orientation and stress level on the dislocation structures generated during primary and steady-state creep in pure aluminium (99.8%). The goal is to characterize features of the microstructure with a statistically sound approach.

## 1. Introduction

The creep behaviour of metallic materials has been studied for about a century (Andrade, 1910 ▸), and numerous articles and reviews have been published on the subject [the papers by Sherby & Burke (1968 ▸), Wilshire & Evans (1985 ▸), Blum (1991 ▸), Ashby (1970 ▸), Arzt & Rösler (1988 ▸), Kassner (1984 ▸), Fernández *et al.* (2016 ▸), Fernández, Bruno *et al.* (2018 ▸), Prager (2000 ▸), Garofalo & Butrymowicz (1966 ▸), Takeuchi *et al.* (1978 ▸), Kassner (2015 ▸) and Evans & Harrison (1979 ▸) represent a sparse selection, to which the reader is referred]. Despite these efforts, hardly any advances have been made on the predictive capabilities of current models to describe the phenomenon.

The broad knowledge of the creep phenomenon accumulated throughout the past decades has led to various models that describe the dependence of the steady-state strain rate, dε/d*t*, on the applied stress, σ. These include power (Sherby & Burke, 1968 ▸), exponential (Nabarro & de Villiers, 1995 ▸; Servi & Grant, 1951 ▸) and even sinh (Ashby, 1970 ▸) dependencies. Interestingly, these models have also been considered at low temperatures (Christian *et al.*, 1964 ▸). The power-law creep equation is the most widely accepted because of the broad experimental basis accumulated (Sherby & Burke, 1968 ▸). However, there is an important limitation: a power-law breakdown at high stress levels is also observed, and the stress exponent, *n*, in this framework increases with σ. In contrast to the power-law equation, the sinh one allows the phenomenon to be described over a wide range of stresses under a unique *n* value (McVetty, 1943 ▸). The sinh equation, which considers an effective diffusion coefficient taking into account the dislocation density, made it possible 40 years ago to describe the creep behaviour of pure aluminium over a range of 21 orders of strain rate magnitude (McVetty, 1943 ▸). However, this equation is largely empiric and does not have a solid physical basis (Christian *et al.*, 1964 ▸). In addition, it has shown several inconsistencies in the description of the secondary creep regime (Evans & Harrison, 1979 ▸). These weaknesses have led scientists to explain the power-law breakdown by modifying the power-law equation (Kassner, 1984 ▸; Fernández, Bruno *et al.*, 2018 ▸; Garofalo & Butrymowicz, 1966 ▸; Takeuchi *et al.*, 1978 ▸; Kassner & Pérez-Prado, 2000 ▸; Fernández, González-Doncel & Garcés, 2020 ▸).

Beyond the well known importance of atom diffusion (Sherby & Burke, 1968 ▸) as a rate-controlling mechanism for dislocation motion, understanding the dislocation dynamics which account for the power-law dependence of dε/d*t* on σ is still a pending task.

Dislocations involved in the creep phenomenon evolve under the action of the applied stress not only individually but also collectively, giving rise to complex dislocation structures (Nye, 1953 ▸; Fernández, Bruno *et al.*, 2018 ▸; Frost & Ashby, 1982 ▸; Shen *et al.*, 2011 ▸; Ishikawa *et al.*, 2002 ▸; Fernández, González-Doncel & Garcés, 2020 ▸). The number of dislocations that produce material deformation (density of mobile dislocation) is usually considered proportional to the applied stress via the Taylor equation (Taylor, 1934 ▸). When attempting to evaluate the role of dislocation in creep deformation, other aspects to consider are that (*a*) the stress fields of dislocations generate attraction or repulsion between them, according to classic dislocation theory (Weertman, 1955 ▸); and (*b*) the dislocation climbing processes, assisted by vacancy diffusion, also play an important role in the case of deformation at elevated temperature.

Recently, a model has been proposed in which the distribution of dislocations generated during the creep process allows the description of the creep behaviour at high stresses without the need for a sinh-type equation (Fernández, González-Doncel, Garcés *et al.*, 2020 ▸; Fernández, González-Doncel & Garcés, 2020 ▸; Fernández *et al.*, 2016 ▸; Fernández, Bruno *et al.*, 2018 ▸). One of the main ideas of the model is that the population of dislocations evolves during creep deformation from an initial disordered arrangement in the early stages of deformation to a well defined cellular structure in the steady state. The model also assumes a fractal arrangement of the cellular structures, which should strongly affect the creep-rate-controlling diffusion process. Thus, these cellular structures would be the basis to justify a possible superdiffusion effect, explaining the increase in strain rate for high values of applied stress (*i.e.* in the power-law breakdown regime). For this reason, it is essential to describe in detail the distribution of stored dislocations during the creep process.

Stored dislocations, which are necessary to maintain the strain compatibility under applied stress, are commonly known as geometrically necessary dislocations (GNDs) (Nye, 1953 ▸; Ashby, 1970 ▸; Hughes *et al.*, 2003 ▸). The global effect of GNDs is generally considered by a net non-zero Burgers vector, which describes the accommodation of a lattice curvature resulting from long-range deformation gradients. Such angular rotation of the lattice is detectable by electron backscatter diffraction (EBSD). On the other hand, statistically stored dislocations (SSDs) are those that accommodate homogeneous deformations by compensating statistical trapping processes (*e.g.* by forming dipoles or tangles). Thus, SSDs have no cumulative effect on the lattice curvature. EBSD is commonly used when examining the effect of the crystal structure and orientation on the microstructural evolution at the micro- and mesoscale (see *e.g.* Wilkinson & Britton, 2012 ▸; Humphreys, 2001 ▸; Barnwal *et al.*, 2017 ▸; Huang *et al.*, 2016 ▸). However, these studies mainly deal with the micromechanical behaviour of metals subjected to forming processes inducing severe deformation, such as cold rolling, whereas the case of high-temperature deformation (creep) of metallic alloys has so far been barely studied.

The aim of the present work is, therefore, to investigate the influence of crystal orientation and stress level on the dislocation structures generated during primary and steady-state creep in pure aluminium (99.8%) by means of Hough-transform-based EBSD and kernel average misorientation (KAM) analysis. The key factor of this study is the post-processing of the acquired EBSD data using a denoising filter to enhance the angular resolution. We will see that such techniques disclose intragranular dislocation structures, enabling us to investigate the way in which the intragranular misorientation varies depending on the applied stress and grain crystallographic orientation.

## 2. Materials and methods

The specimens used in this investigation were machined out of a hot extruded pure Al (99.8%) bar of 7 mm diameter. The imposed extrusion temperature was 800 K and the resulting pressure was 400 MPa. This material has a yield stress of 48 MPa at room temperature.

### 2.1. Crystallographic texture

X-ray texture measurements were carried out using a Siemens D5000 diffractometer by means of the Schulz reflection method. The type of X-ray radiation was Cu *K*α (β filtered). The pole figures 〈111〉, 〈200〉, 〈220〉 and 〈311〉 were measured using sliced samples, where the surface exposed to the X-ray radiation was normal to the extrusion axis. The surface polishing protocol was similar to that used for the preparation of microscopy samples (see Section 2.3). In addition, the inverse pole figure of the extrusion axis was computed.

### 2.2. Creep testing

The creep tests were conducted at constant stress, ensured by means of an Andrade cam that compensates for the decrease in sample section as creep tensile deformation progresses. Cylindrical tensile samples were machined with the tensile axis direction parallel to the extrusion direction. The samples had threaded heads, a 10 mm gauge length and a cross section of 3 mm diameter. The elongation was recorded as a function of time by means of two digital strain gauges (SOLARTRON model DP/5/S) with a sensitivity of 0.2 µm. The sample clamping system and the strain gauges allowed suppression of the contribution of the machine and the grips from the sample creep strain. The tests were conducted at 573 K and two different stress levels: 21 and 29 MPa. As will be seen, these two values lie in the power-law and the power-law breakdown regimes, respectively. A heating rate of 100 K h^−1^ was used from room temperature to 573 K, followed by 1 h soaking time. The samples were strained up to 0.0025. In order to study the dislocation structure achieved at each strain level, the samples were rapidly cooled (to room temperature, RT) by using an air jet while still applying the stress (21 or 29 MPa). This process took around 3 min, which meant a cooling rate of around 6000 K h^−1^. Thus, it is assumed that, even if some diffusion still occurs during and after cooling, the samples used for the EBSD analysis retained dislocation structures reasonably similar to those developed during creep deformation (Caillard & Martin, 1987 ▸; Yavari *et al.*, 1981 ▸).

### 2.3. EBSD experiments

The EBSD measurements were performed using a field-emission gun scanning electron microscope (LEO 1530VP, Zeiss, Germany) operating at an acceleration voltage of 20 kV and with a probe current of 7–8 mA in high-pressure mode. The working distance was 16–17 mm and the diaphragm aperture 120 µm. A Bruker Nano e-Flash HD 5030 detector and the *ESPRIT* software package were used for the EBSD-pattern acquisition and evaluation, respectively. The evaluation of orientations was based on 10 × 10 binned EBSD patterns (*i.e.* the measured raw patterns were 8-bit greyscale images containing 160 × 120 pixels). During Hough-transform-based pattern indexing, a combination of static and dynamic background correction available in the software was used.

Sample preparation required the specimens to be embedded in resin before undertaking a reduction of the diameter via the grinding of the samples down to a diameter of ∼1.5 mm. Subsequently, a metallographic standard preparation (grinding with SiC papers and polishing using diamond slurries down to 3 µm) was performed. Special care was taken during the final polishing (using 0.02 µm colloidal silica suspension for 10 min) to remove any residual deformation introduced during the preceding preparation steps.

EBSD maps were acquired at two different magnifications: at ×250 (step size = 2.37 µm, leading to an investigated area of 1213 × 910 µm), to obtain a mesoscale overview of the grain structure; and at ×2000 (step size = 0.29 µm, area of 152 × 114 µm), to resolve the dislocation structures at the microscale. The step size at ×2000 was chosen as a good compromise between obtaining a good spatial resolution, keeping the noise measurement low, and the need for measurement areas that are statistically relevant. In addition, EBSD maps were acquired on both gauge and grip regions of the crept samples; this allowed separation of the effect of the applied stress from that of extruding + annealing. Note that only the grip of the sample tested at 21 MPa was analysed, as it was assumed that the heat treatment conditions at the grips were the same for both applied stress levels.

### 2.4. Data analysis

The KAM parameter is defined as the average misorientation angle between the crystallographic orientation at a given point and those of the surrounding points. Herein, the KAM has been determined at the two magnifications used. The meaning of the KAM in terms of microstructural characteristics is strongly dependent on the magnification at which this parameter is obtained. This aspect will be discussed in detail in Section 3. The literature has shown that this local misorientation parameter provides a good estimate of the microstructural deformation (Hughes *et al.*, 1997 ▸; Wright *et al.*, 2011 ▸; Nye, 1953 ▸). Therefore, it can also be used to evaluate the intragranular plastic deformation caused by creep (Wright *et al.*, 2011 ▸). The KAM is also commonly considered to evaluate the severity of the deformation: a higher content of dislocations leads to higher angles of misorientation (Muránsky *et al.*, 2019 ▸; Rui *et al.*, 2019 ▸).

The 2D Hough-transform indexing route of Kikuchi-band positions is conventionally adopted in automated commercial software packages for EBSD analysis. The main disadvantage of this route is its limited angular resolution (typically 0.5–1°; Ram *et al.*, 2015 ▸). This limitation implies that very low angle dislocation structures are not detectable. Whereas the amount of noise in ESBD maps may seem negligible for the analysis of the grain structure, this noise is significantly amplified if the raw EBSD data are used for subsequent calculations of misorientations (Ram *et al.*, 2015 ▸). Nevertheless, the accuracy of orientation maps can be significantly increased (up to 0.05°) by using denoising filters to enhance standard Hough-transform indexed data (Hielscher *et al.*, 2019 ▸).

The *MTEX* toolbox (version *mtex-5.6.0*; Bachmann *et al.*, 2011 ▸) installed on MATLAB was used for the post-processing of the raw EBSD data, where two criteria were used: (i) the misorientation between neighbouring grains must be greater than 5°; and (ii) each grain must contain at least 5 data points. In addition, the orientation data were denoised by applying a total-variation-based filter adapted from image analysis toolboxes (Hielscher *et al.*, 2019 ▸). Applying this denoising filter before computing the KAM (where only the first-order neighbours are considered) is sufficient to resolve details of dislocation structures without generating artificial structures (*i.e.* the subgrain boundaries remain sharp whereas regions containing salt-and-pepper noise become smooth).

## 3. Results

### 3.1. Microstructure

**Figure 1:**
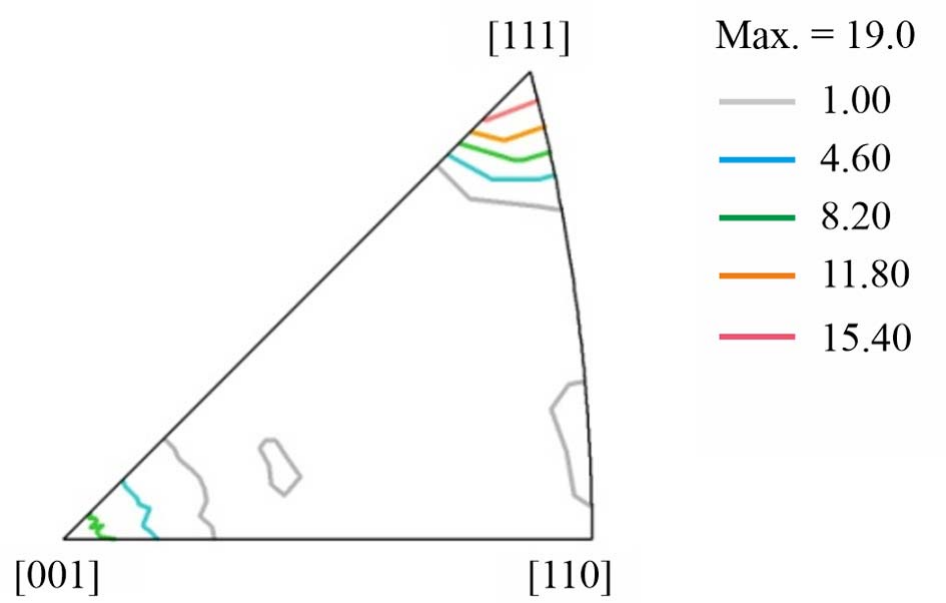
X-ray inverse pole figure (IPF) for the extrusion axis.

The global crystallographic texture shows a 〈111〉 + 〈001〉 fibre texture (with the fibre axis parallel to the extrusion/tensile direction; Fig. 1 ▸) typical of extruded face-centred cubic metals.

The grain size was obtained from EBSD maps by calculating the area weighted mean of the equivalent surface diameter. Such a diameter belongs to the circle with equivalent surface area, Ø_eq_ = 2(*A*/π)^1/2^. Only the grains fully contained in the images were considered in the calculation (*i.e.* the grains in contact with the borders of the map were discarded). Four EBSD maps acquired at ×250 magnification yielded an average grain size of 169 ± 62 µm.

### 3.2. Creep tests

**Figure 2:**
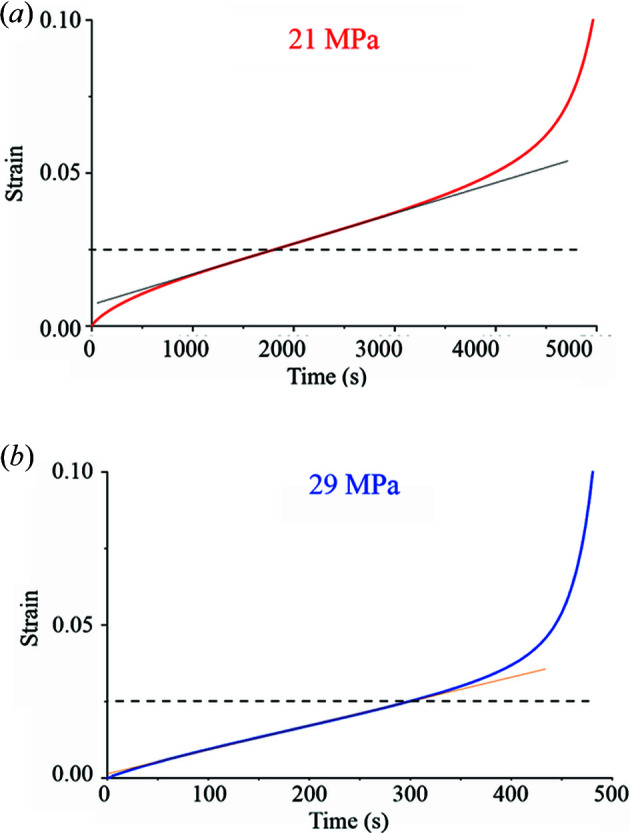
Plots showing the correspondence of the 0.025 strain to the secondary creep regime at 573 K for the two investigated stress levels. In case (*b*), the strain rate achieved lies in the power-law breakdown regime.

The complete creep curves (strains versus time, for tests conducted until failure) at the two investigated stress levels are shown in Fig. 2 ▸. The dashed lines indicate the strain level (0.025) at which subsequent creep tests were interrupted to produce the samples investigated herein. In both cases, the strain remains within the steady state. The strain rate corresponds to the power-law regime in the case of the test conducted at 21 MPa and to the power-law breakdown case at 29 MPa.

### 3.3. EBSD orientation maps and KAM analysis

#### 3.3.1. Samples at ×250 magnification

**Figure 3:**
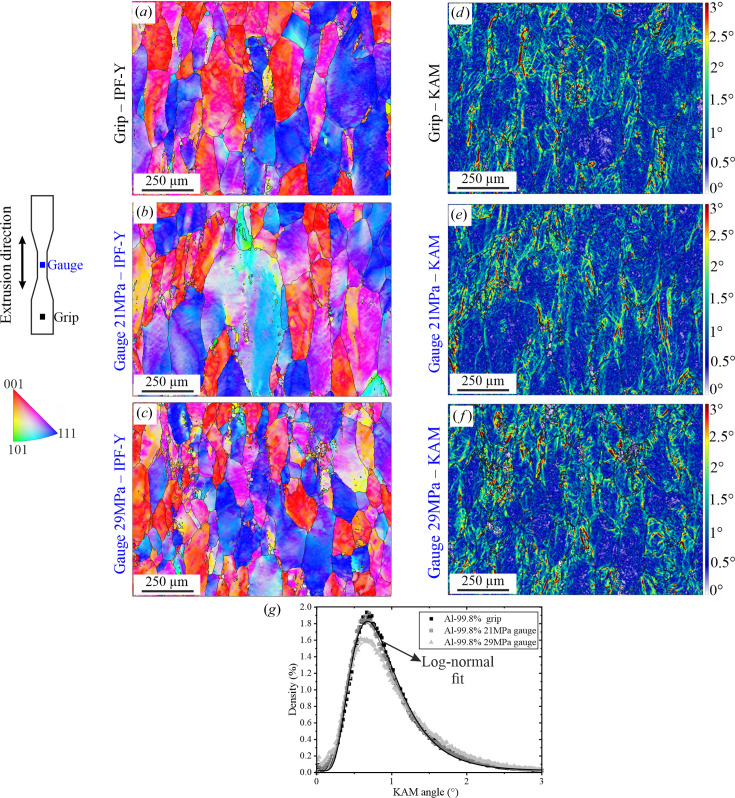
EBSD results acquired at ×250 and their analysis: (*a*) EBSD orientation map (IPF-Y; parallel to the loading direction) acquired on the grip; (*b*) EBSD map acquired on the gauge of the sample tested at 21 MPa; (*c*) EBSD map acquired on the gauge of the sample tested at 29 MPa. (*d*) KAM-angle map of the grip. (*e*) KAM-angle map at 21 MPa. (*f*) KAM-angle map at 29 MPa. (*g*) KAM-angle histograms and corresponding log-normal fits.

Figs. 3 ▸(*a*)–3 ▸(*c*) show the orientation maps of the two regions (grip or gauge) in the investigated specimens, acquired at ×250 magnification; these maps are colour-coded according to the inverse pole figure relative to the *Y* axis (*i.e.* the extrusion and loading direction). In these images, the grains show a predominance of blue 〈111〉 and red 〈001〉 colouring (as expected from Fig. 1 ▸), and continuous colour gradients (*e.g.* from blue to cyan) are observed within the majority of the grains, implying intragranular misorientations. Figs. 3 ▸(*d*)–3 ▸(*f*) show the KAM-angle maps derived from the denoised orientation data. No significant differences are observed among the three KAM-angle maps: they all exhibit predominance of blue colouring (corresponding to 0.5–1° misorientation). Nevertheless, several locations of increased misorientation angles (green to red colouring, corresponding to 1.5–3°) are observed, typically in the vicinity of grain boundaries.

The KAM-angle histograms corresponding to the three regions are shown in Fig. 3 ▸(*g*). The misorientation values mainly vary in the 0.2–2.5° range. The misorientation distribution is well represented by a log-normal function for both crept (gauge regions) and solely annealed (*i.e.* grip region) conditions. The mean misorientation value, μ, and the standard deviation of all distributions are given in Table 1 ▸. Interestingly, the distributions resulting from the grip and gauge tested at 21 MPa are almost identical. The distribution of the 29 MPa sample shows an increase of μ of about 0.03°. Since it is below the standard deviation, this increase is most likely induced by local variations in the studied area rather than by a general trend. This point is confirmed by the qualitative analysis of Figs. 3 ▸(*d*)–3 ▸(*f*): no substantial microstructural differences can be observed between the solely annealed sample and the crept samples.

Note that the standard deviation given in Table 1 ▸ is not intended to represent a variation of the measured value, and hence an experimental uncertainty; it is rather a measure of the inhomogeneity of the spatial distribution of the observed substructures. In this case, the results show that the spread of the misorientation increased from minimum at the grip to maximum at the gauge at 29 MPa. Also, we could remark that, due to the high mobility of dislocations at 573 K, some degree of diffusion-driven recovery (*e.g.* reduction in the misorientation level as compared with the original extruded material) is expected to occur in the sample grips during the creep tests as a result of the annealing process.

#### 3.3.2. Samples at ×2000 magnification

**Figure 4:**
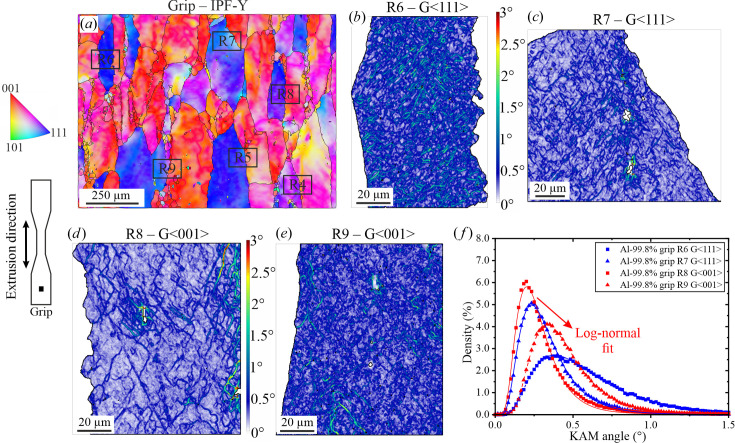
(*a*) EBSD orientation map of the grip region at ×250 showing the location of the different regions analysed at ×2000. KAM-angle maps for (*b*) the blue 〈111〉 grain located in R6, (*c*) the blue 〈111〉 grain located in R7, (*d*) the red 〈001〉 grain located in R8 and (*e*) the red 〈001〉 grain located in R9. (*f*) KAM-angle histograms and the corresponding log-normal fits.

*Grip region: extruded+annealed material*. Fig. 4 ▸(*a*) shows the orientation map at ×250 magnification that was used as reference to localize the regions (R*i*, *i* = 4 to 9) to be analysed at ×2000 magnification. Such regions extend over different grains, so that the subregions are indicated by the grain to which they belong (*e.g.* R*i*-G〈*hkl*〉). The most representative of the analysed grains are shown in Figs. 4 ▸(*b*)–4 ▸(*e*) (the results of the remaining grains are given in the supporting information). Grain R6-G〈111〉 corresponds to the highest bound (*i.e.* the grain with the highest observed misorientation) and grain R8-G〈001〉 the lowest one. The intragranular dislocation structures are visible at ×2000 magnification and exhibit misorientations principally between 0.5 and 1.5° (*i.e.* from blue to green). For the sake of simplicity, these dislocation structures are named substructures hereafter (Bay *et al.*, 1992 ▸; Liu & Hansen, 1995 ▸).

The main difference between the grains shown in Figs. 4 ▸(*b*)–4 ▸(*e*) corresponds to the amount of substructure: the grains shown in Figs. 4 ▸(*b*) and 4 ▸(*e*) are observed to contain high-dislocation-density substructure walls (dark-blue to red colouring, *i.e.* 0.5–3° range), as opposed to the grains shown in Figs. 4 ▸(*c*) and 4 ▸(*d*), which exhibit a predominance of lower misorientations (light-blue colouring, *i.e.* 0.2–0.3° range). In other words, the average size of the substructures is higher in Fig. 4 ▸(*c*) and 4 ▸(*d*). This difference in substructure density induces a distinct upwards shift in the log-normal distributions [Fig. 4 ▸(*f*)], where the substructure low-density grains have μ values in the 0.25–0.3° range, while those with high density have μ values in the 0.4–0.5° range (see Table 2 ▸).

The standard deviation shown in Table 2 ▸ indicates that grains-〈001〉 tend to have a lower spread of the misorientation.

**Figure 5:**
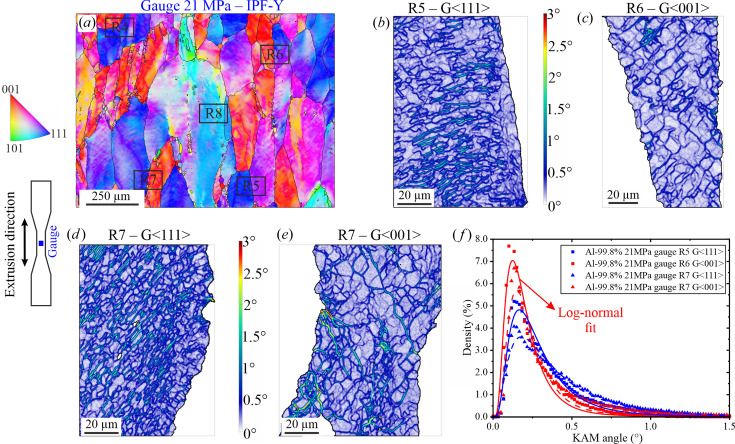
(*a*) EBSD orientation map of the 21 MPa gauge region at ×250 of the sample tested at 21 MPa showing the location of the different regions analysed at ×2000. KAM-angle maps for (*b*) the blue 〈111〉 grain located in R5, (*c*) the red 〈001〉 grain located in R6, (*d*) the blue 〈111〉 grain located in R7 and (*e*) the red 〈001〉 grain located in R7. (*f*) KAM-angle histograms and the corresponding log-normal fits.

*Gauge region: crept material at 21 MPa*. Figs. 5 ▸(*a*)–5 ▸(*e*) show the location and KAM results of some of the grains investigated at ×2000 magnification in the gauge of the sample tested at 21 MPa. As above, only the most representative grains are given as an example [Figs. 5 ▸(*b*)–5 ▸(*e*)]. In comparison with the grip region, better defined subgrains (*i.e.* cellular substructures with overall thicker dislocation walls) are formed after creep. Also, the subgrain interior has recovered, showing a lower level of misorientation (colouring close to white 0–0.1° instead of light blue for the grip). This phenomenon shifts the KAM distribution to lower values [Fig. 5 ▸(*f*)]. Again, there are grains showing a higher density of subgrains [Figs. 5 ▸(*b*) and 5 ▸(*d*)] than others [Figs. 5 ▸(*c*) and 5 ▸(*e*)], although the difference is smaller in this case than in the grip. This tendency is confirmed by the narrower variation in mean μ values among the grains (∼0.2–0.3°). Note that for the gauge regions the log-normal fit does not describe the KAM distribution of the investigated grains as well as for the grip regions, but its parameters are used for the sake of comparison. The level of misorientation in the cell walls resulting from creep is similar to that observed in the substructure walls (0.5–3° range); however the recovery in the subgrains’ interior leads to higher values of the standard deviation (now between ∼0.6 and 0.8°; *i.e.* an average 0.1° increase). Furthermore, the standard deviation shown in Table 3 ▸ indicates that grains-〈001〉 tend to have a lower spread of the misorientation.

**Figure 6:**
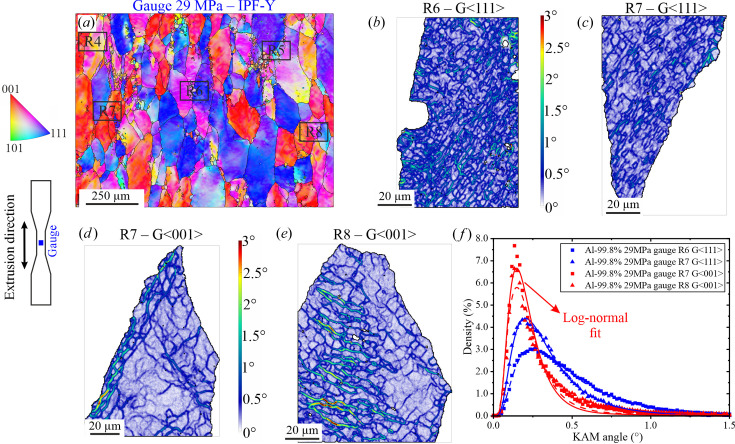
(*a*) EBSD orientation map at ×250 of the sample tested at 29 MPa gauge region showing the location of the different regions analysed at ×2000. KAM maps for (*b*) the blue 〈111〉 grain located in R6, (*c*) the blue 〈111〉 grain located in R7, (*d*) the red 〈001〉 grain located in R7 and (*e*) the red 〈001〉 grain located in R8. (*f*) KAM histograms and corresponding log-normal fits.

*Gauge region: crept material at 29 MPa*. Figs. 6 ▸(*a*)–6 ▸(*e*) show the location and KAM results of some of the grains investigated at ×2000 magnification in the gauge of the sample tested at 29 MPa. The subgrain structures are very similar to those observed in the 21 MPa sample. The similarity is also maintained in terms of variability of KAM distributions [Fig. 6 ▸(*f*)], as well as for μ and the standard deviation parameters of the log-normal fit (Table 4 ▸). Furthermore, at 29 MPa the 〈111〉 grains also tend to possess higher levels of misorientation when compared with 〈001〉 grains. From a qualitative point of view, it is observed that the subgrain spatial distribution has an increased heterogeneity in the 29 MPa sample (*i.e.* higher variability of the size of the subgrains within one grain and increased tortuosity).

The standard deviation shown in Table 4 ▸ also indicates that grains-〈001〉 tend to have a lower spread of the misorientation similar to that observed at 21 MPa (Table 3 ▸).

**Figure 7:**
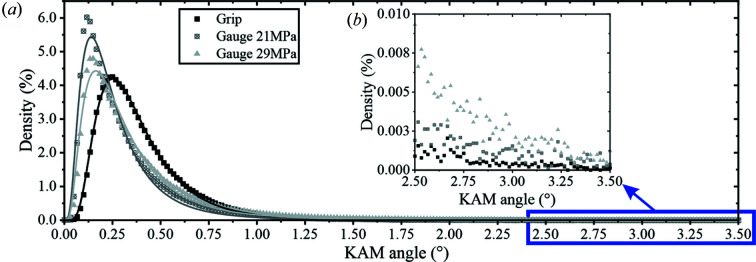
(*a*) KAM histograms showing misorientation distributions at ×2000, where the data of all analysed regions for each condition are summed. (*b*) Inset of the preceding KAM histogram in the region between 2.5 and 3.5°.

Fig. 7 ▸(*a*) shows the KAM distribution resulting from combining the data acquired in all the regions shown in each EBSD map of Fig. 4 ▸(*a*), Fig. 5 ▸(*a*) and Fig. 6 ▸(*a*). The increased statistics confirm what was already observed using individual grains: the log-normal fit matches better the dislocation structures in the extruded + annealed condition than those in the two crept conditions. Also, the mean (μ) misorientation is lower for the creep conditions (0.218° at 21 MPa and 0.264° at 29 MPa against 0.337° for the grip). Importantly, these results allow us to clearly observe that the 29 MPa condition accumulates a higher amount of misorientation in the 1–3° range. Specifically, the density of misorientation at 2.5° in the 29 MPa is double that of the 21 MPa condition [Fig. 7 ▸(*b*)].

Note that the overall misorientation mean (μ) at ×2000 (∼0.2–0.6°) for all the investigated conditions (Fig. 4 ▸, Fig. 5 ▸ and Fig. 6 ▸) is lower than that obtained when analysing the conditions at ×250 (∼0.8°, Fig. 3 ▸). This is an effect of the low spatial resolution at ×250, where the chosen step size (2.37 µm) is larger than the underlying dislocation structures. Consequently, the binning of several substructures/subgrain structures in one pixel leads to an artificial increase of the calculated misorientation (Wright *et al.*, 2011 ▸).

## 4. Discussion

Typically, in materials with high stacking-fault energy (SFE) such as pure aluminium, the storage of dislocations in geometrically necessary boundaries (GNBs) is preferred over SSDs (Hansen *et al.*, 2001 ▸). Moreover, in pure aluminium the mobility of dislocations at high temperature is very high. During a creep test, this high mobility allows a large number of dislocations to disappear by recombination (recovery). It is well known that the high mobility, fostered by the high number of available vacancies, leads to the formation of 3D dislocation-free cells that are enclosed by dislocation walls (Kuhlmann-Wilsdorf & Hansen, 1991 ▸; Bay *et al.*, 1992 ▸; Fernández, González-Doncel, Garcés *et al.*, 2020 ▸). Such a dislocation structure will evolve during the primary creep regime, forming subgrain boundaries until the final structure is completed in the secondary regime.

**Figure 8:**
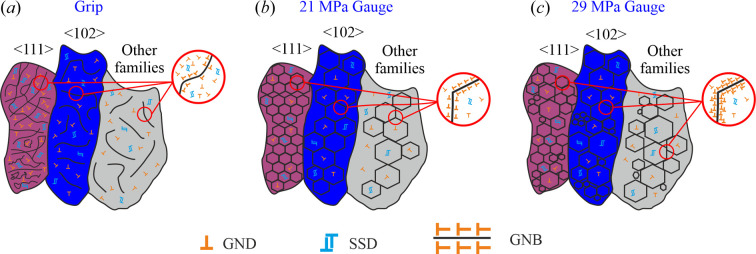
Schematic illustrations summarizing the findings in the case of (*a*) the grip, (*b*) the gauge at 21 MPa and (*c*) the gauge at 29 MPa. For details see the text.

We observe that the subgrains in the secondary creep regime (gauge regions) are in general more ordered than the substructures after annealing (in the grip); this is sketched in Fig. 8 ▸. This ordering would lead to lower misorientation levels (described by KAM) in the cell interior of the crept samples compared with the substructure interior in the solely extruded+annealed material. At this point it is important to remember that dislocation movement during creep deformation is generally thought to be dominated by diffusive processes, such as lattice self-diffusion, possible contributions from pipe diffusion, grain boundary diffusion and/or increased diffusivity caused by excess vacancy generation from dislocation motion. Also, it is assumed that the changes in the subgrain structures occurring during the steady/secondary creep are minimal (*i.e.* the dislocation density remains constant) (Caillard & Martin, 1987 ▸).

Another important finding is the fact that testing at 29 MPa increases the inhomogeneity of subgrain structures [Figs. 8 ▸(*b*) and 8 ▸(*c*)]. It is likely that the increase in the average misorientation at the subgrain walls in the sample tested at 29 MPa (Fig. 7 ▸) results from the higher dislocation density created during the primary creep, whereby a smaller amount of dislocation recombination would be induced during the secondary stage (since inhomogeneous strain fields are expected to hamper such recombination). Moreover, since an applied stress of 29 MPa lies in the power-law breakdown region, where pipe diffusion dominates (Nabarro & de Villiers, 1995 ▸), the increased wall misorientation in the 29 MPa state would be explained by the ability of the cellular structures to carry more dislocations.

**Figure 9:**
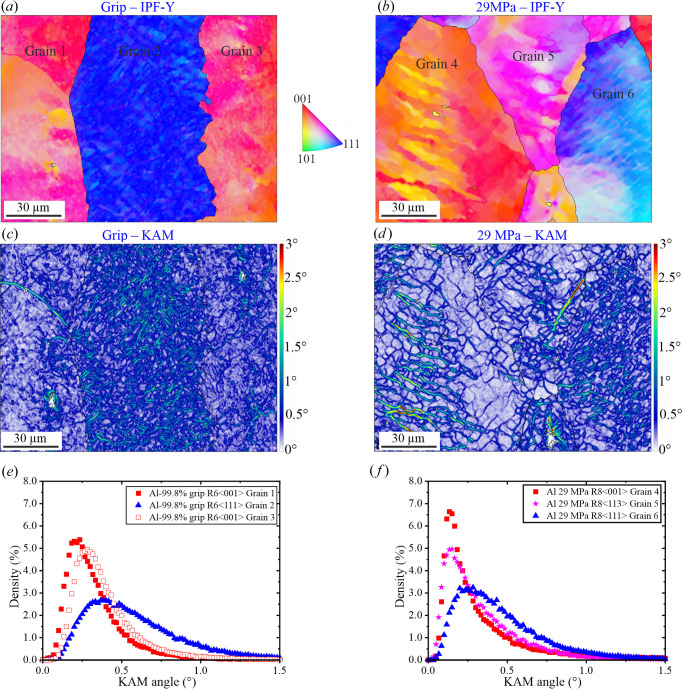
EBSD orientation map at ×2000 of (*a*) the grip (extruded+annealed condition) and (*b*) the gauge region of the sample tested at 29 MPa; (*c*) and (*d*) are the respective KAM maps; and (*e*) and (*f*) are the corresponding KAM histograms of each individual grain.

Depending on the conditions (extruded versus crept) and the crystallographic orientation of grains, significant differences are found in the distribution and density of cellular/subgrain structures (see Fig. 9 ▸). In the grip, comparison among grains indicates that during the hot extrusion process the grains exhibiting 〈111〉 crystallographic orientations tend to develop, on average, denser substructures than the 〈100〉 grains. Such substructures resulting from the extrusion process are expected to undergo some degree of diffusion-driven recovery during the annealing process occurring at the grip during the creep test. Note that the same inhomogeneity in subgrain structure density as a function of crystal orientation is observed in the crept material [the case at 29 MPa is shown in Figs. 9 ▸(*b*), 9 ▸(*d*) and 9 ▸(*f*)].

A plausible, though qualitative, explanation of the observed differences in Fig. 9 ▸ rests on the contribution of the intergranular stress of individual grains developed during the extrusion process. For an extruded 2014 aluminium alloy containing a similar macroscopic texture to our samples (〈111〉 + 〈100〉), it was observed that 〈111〉 grains tend to develop a tensile axial stress while 〈100〉 grains possess compressive axial stress (Fernández, Ferreira-Barragáns *et al.*, 2018 ▸). Consequently, we can safely assume that the severe strain imposed during extrusion induces an intergranular stress state that is inherited during creep. Since it is driven by compatibility conditions, such intergranular stress is not completely relieved during creep. Therefore, during the creep test, grains with 〈111〉 orientation experience a total stress that is the sum of the applied and the intergranular stress, while the 〈100〉 grains would undergo the applied stress diminished by the compressive intergranular stress. In summary, it is realistic to assume that the ‘actual’ uniaxial stress that individual grains experience during creep is higher in the 〈111〉 than in the 〈100〉 grains. Using Taylor’s relationship between subgrain size and creep stress (Blum, 1991 ▸), it is readily concluded that 〈111〉 grains, when tested at higher stress than the 〈100〉 ones, develop finer cell sizes than the 〈100〉 grains (see Figs. 4 ▸, 5 ▸ and 6 ▸).

When comparing our results with direct evidence of dislocation structures as obtained by transmission electron microscopy (TEM) (see *e.g.* Fernández, Bruno *et al.*, 2018 ▸), we see that with the use of EBSD we could investigate a field of view much larger than that achievable by TEM. While TEM would be necessary to image single dislocation walls and tangles, the statistical significance of the present EBSD–KAM observations is much higher and allows us to draw conclusions based on higher statistics, as well as to study the influence of grain orientation and grain environment. Moreover, this work paves the way to the investigation of creep performance as a function of crystallographic texture.

## 6. Summary and conclusions

The dislocation structures formed in an extruded pure Al (99.8%) material after creep tests at two different stress levels have been investigated by means of electron backscatter diffraction and kernel average angular misorientation analysis. In this work we could observe grain (dislocation) substructures/subgrains, as well as intragranular misorientations. Moreover, it was possible to correlate the evolution of such dislocation structures with the grain crystallographic orientation.

We can summarize the principal findings as follows:

(i) KAM maps at high magnification (×2000) allow a detailed investigation of dislocation structures in pure aluminium, as they clearly distinguish microstructures before and after creep and in differently oriented grains. In other words, KAM is proven to be an extremely valuable tool for microstructural investigation at the mesoscale (from a few tenths of a millimetre to a few millimetres).

(ii) Correspondingly, we developed a strategy to analyse different stages of creep deformation by EBSD mapping at low resolution and refinement at high resolutions.

(iii) The extruded+annealed material contains highly intertwined substructures, which recombine to form well defined subgrains after creep. Such random-distributed structures formed during extrusion remain after an annealing process.

(iv) The subgrain structures are more homogenously distributed over the grain’s areas at 21 MPa than at 29 MPa. In addition, the level of misorientation within grains increases at 29 MPa when compared with 21 MPa.

(v) It is proposed that the inhomogeneity in the distribution of subgrain structures among grains is mainly driven by the crystal orientation, in both the 21 MPa and 29 MPa conditions, due to the effect of intergranular stresses.

Still more evidence needs to be produced to corroborate the scenarios set forth in this work, and the findings need to be proven on other materials (*e.g.* Al alloys). The combination of macroscopic (creep tests), mesoscopic (EBSD–KAM and X-ray diffraction) and microscopic (scanning and transmission electron microscopy) characterization techniques will allow further insight to be gained into creep deformation mechanisms.

## Supplementary Material

Three additional figures showing KAM results and their corresponding histograms to provide further statistics for the results and tendencies described in the article. DOI: 10.1107/S1600576722005209/te5088sup1.pdf

## Figures and Tables

**Table 1:** Parameters of the log-normal distribution at ×250 magnification of the KAM-angle histogram fits in Fig. 3 ▸(*g*)

	Mean, μ	Standard deviation
Grip MPa	0.836	0.458
Gauge 21 MPa	0.821	0.473
Gauge 29 MPa	0.869	0.536

**Table 2:** Parameters of the log-normal distribution fits used to describe the KAM-angle histograms in Fig. 4 ▸(*f*)

	Mean, μ	Standard deviation
R6-G〈111〉	0.53	0.57
R7-G〈111〉	0.30	0.50
R8-G〈001〉	0.25	0.48
R9-G〈001〉	0.41	0.44

**Table 3:** Parameters of the log-normal distribution fits used to describe the KAM-angle histograms in Fig. 5 ▸(*f*)

	Mean, μ	Standard deviation
R5-G〈111〉	0.25	0.64
R6-G〈001〉	0.18	0.60
R7-G〈111〉	0.33	0.77
R7-G〈001〉	0.21	0.56

**Table 4:** Parameters of the log-normal distribution fits used to describe the KAM histograms in Fig. 6 ▸(*f*)

	Mean, μ	Standard deviation
R6-G〈111〉	0.41	0.70
R7-G〈111〉	0.30	0.62
R7-G〈001〉	0.20	0.56
R8-G〈001〉	0.20	0.52
