# Supplementary material for: Dislocation substructures in pure aluminium after creep deformation as studied by electron backscatter diffraction
Source: J Appl Crystallogr. 2022 Jul 5;55(Pt 4):860–9. doi: 10.1107/S1600576722005209 (PMC9348881; doi:10.1107/S1600576722005209)

JOURNAL OF APPLIED CRYSTALLOGRAPHY

Volume 55 (2022)

Supporting information for article:

Dislocation substructures in pure aluminium after creep deformation as studied by the electron backscatter diffraction technique

Itziar Serrano-Munoz, Ricardo Fernández, Romeo Saliwan-Neumann, Gaspar González-Doncel and Giovanni Bruno

Fig. S1. (*a*) EBSD orientation map of the grip region at x250 magnification showing the location of the different regions analysed at x2000 magnification. KAM-angle maps for: (*b*) the blue-<111> grain located in R4, (*c*) the magenta-<113> grain located in R4, (*d*) the blue-<111> grain located in R5, (*e*) the red-<001> grain located in R5, (*f*) the red-<001>-1 grain located in R6, (*g*) the red-<001>-2 grain located in R6, (*h*) the red-<001> grain located in R7, (*i*) the blue-<111> grain located in R8, and (*j*) the blue-<111> grain located in R9. (*k*) KAM-angle histograms of the preceding grains.

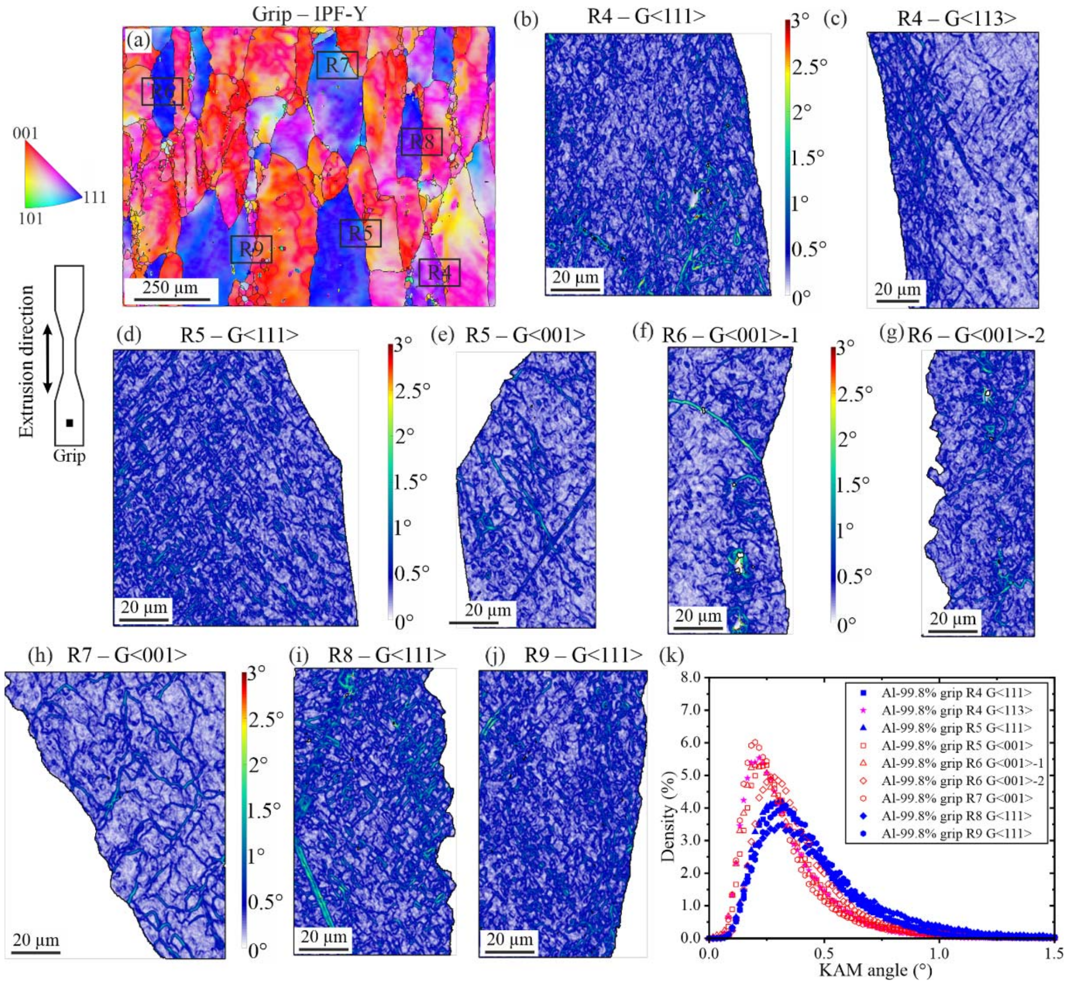

Fig. S2. (*a*) EBSD orientation map of the 21 MPa gauge region at x250 magnification showing the location of the different regions analysed at x2000 magnification. KAM-angle maps for: (*b*) the red-<001> grain located in R4, (*c*) the blue-<111>-1 grain located in R4, (*d*) the blue-<111>-2 grain located in R4, (*e*) the magenta-<113> grain located in R5, (*f*) the blue-<111> grain located in R6, (*g*) the cyan-<122>- grain located in R8, and (*h*) the light-blue-<344> grain located in R8. (*i*) KAM-angle histograms of the preceding grains.

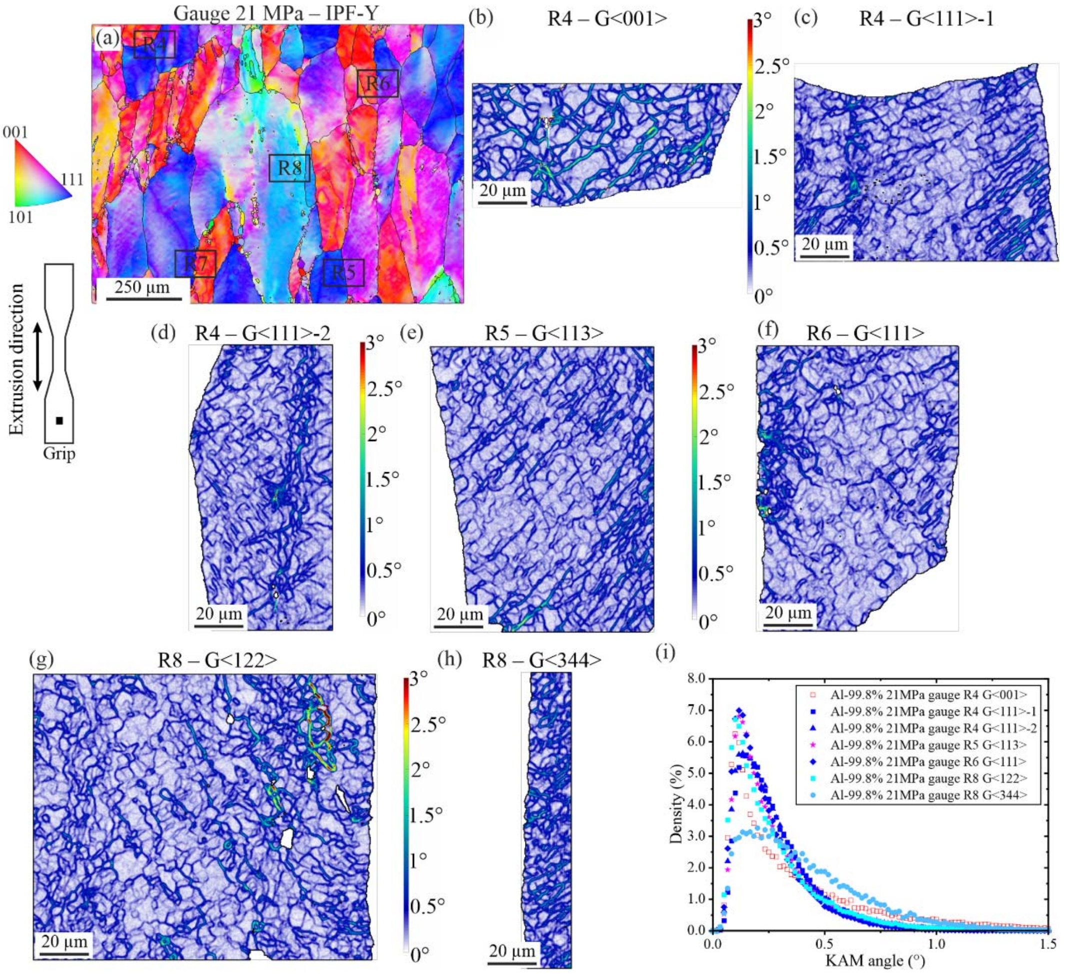

Fig. S3. (*a*) EBSD orientation map of the 29 MPa gauge region at x250 magnification showing the location of the different regions analysed at x2000 magnification. KAM maps for: (*b*) the red-<001>-1 grain located in R4, (*c*) the magenta+yellow<113>+<012> grain located in R4, (*d*) the magenta-<113>-1 grain located in R4, (*e*) the magenta-<113>-2 grain located in R4, (*f*) the blue-<111> grain located in R5, (*g*) the magenta-<113> grain located in R5, (*h*) the magenta-<113> grain located in R6, (*i*) the red-<001> grain located in R7, and (*j*) the blue-<111> grain located in R8. (*k*) KAM-angle histograms of the preceding grains.

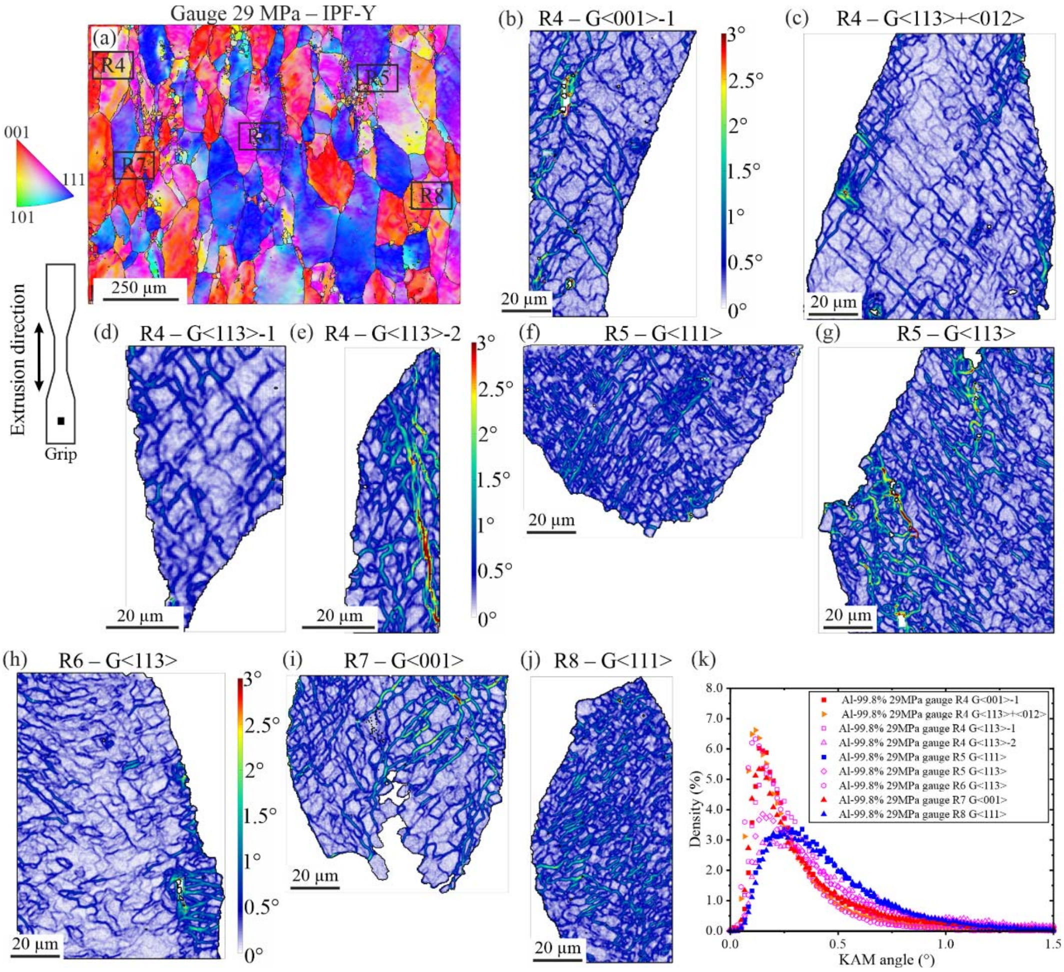

Supplement: Supplementary file 1 [file j-55-00860-sup1.pdf]
